# Screening *Brassica rapa* for broad-spectrum resistance to Turnip mosaic virus

**DOI:** 10.1270/jsbbs.24015

**Published:** 2024-08-27

**Authors:** Ainan Tian, Masaya Yamamoto, Hideki Takahashi, Hiroyasu Kitashiba

**Affiliations:** 1 Graduate School of Agricultural Science, Tohoku University, 468-1 Aramaki Aza Aoba, Aobaku, Sendai, Miyagi 980-8572, Japan

**Keywords:** *Brassica rapa*, turnip mosaic virus (TuMV), plant resistance, broad-spectrum resistance

## Abstract

Turnip mosaic virus (TuMV) poses a major threat to *Brassica* crops like Chinese cabbage, causing significant economic losses. A viable and effective strategy to manage such diseases is by improvement of genetic-based viral resistance. To achieve this, it is important to have detailed and wide-ranging genetic resources, necessitating genetic exploration. To identify useful TuMV resistant genetic resources, we screened geographically and genetically diverse resources including over 90 accessions from the Tohoku Univ. *Brassica* Seed Bank against eleven TuMV isolates phylogenetically classified into five clusters. Two USA accessions exhibited no or only slight symptoms with no virus protein detected in virus-inoculated and non-inoculated upper leaves, suggesting an extreme resistance to all tested TuMV isolates. Through sequencing and dCAPS analysis of eukaryotic translation initiation factor (eIF4E/eIFiso4E) in the 95 *B. rapa* accessions, several amino acid substitutions were observed on the dorsal surface and cap-binding sites of eIF4E/eIFiso4E proteins, with three of them significantly associated with resistance/susceptibility responses. When exploring co-infection using TuMV and cucumber mosaic virus (CMV), the TuMV susceptible accession died, but TuMV resistance was retained in the TuMV resistant accession. It suggested that the broad-spectrum resistance in the two USA accessions is a highly valuable resources for *Brassica* breeding.

## Introduction

The genus *Brassica* is a member of the Brassicaceae family and includes major cultivated vegetables and oil crops. Among the economically important crops belonging to the *B. rapa* species are turnips, Chinese cabbage, pak choy and oilseed crops such as yellow sarson and brown sarson. *B. oleracea* includes cabbage, broccoli, cauliflower, and kale, while *B. napus* is grown mainly as an oil-seed crop. *B. juncea*, *B. carinata*, and *B. nigra* are cultivated as spice-seed plants and oilseed crops. The closely related cultivated species, *Raphanus sativus*, is also a member of the Brassicaceae family, with its roots and capsules used as an edible vegetable. These crops are distributed and cultivated throughout the world, forming a vital source of food and economic value.

Diseases are major threats to the cultivation of these *Brassica* and *Raphanus* vegetables and crops. For example, fungal diseases such as clubroot (caused by *Plasmodiophora* *brassicae*), Fusarium wilt (*Fusarium oxysporum*), and Black spot (*Verticillium dahlia*), or bacterial diseases such as black rot caused by *Xanthomonas campestris* are serious problems (reviewed by Liu *et al.* 2013, Niikura 2017, Xiaona *et al.* 2017). To control these diseases, developing resistant varieties/cultivars is an effective, importantly, and environmentally friendly approach. To achieve this objective, extensive research efforts have been undertaken, including screening for resistant genetic resources, identification of resistance loci, and gene isolation (Liu *et al.* 2013, Niikura 2017, Xiaona *et al.* 2017). Additionally, efforts to identify useful DNA markers and apply these genes to practical breeding studies are currently underway.

Viral diseases also cause severe damage to cruciferous vegetables. Both TuMV (*Turnip mosaic virus*) and CMV (*Cucumber mosaic virus*) cause significant damage to *Brassica* and *Raphanus* vegetables and crops worldwide (Walsh *et al.* 1999, Yu *et al.* 2018). TuMV is a member of the *Potyviridae* family with a positive sense single-stranded RNA genome of about 10 kb encoding for ten proteins (P1, HC-Pro, P3, 6K1, CI, 6K2, VPg, NIa, NIb, and CP) (Yasaka *et al.* 2017). Based on a phylogeographic genomic study, TuMV is genetically divided into orchis, Iranian, basal-B, basal-BR, Asian-BR, and world-B groups (Kawakubo *et al.* 2021). TuMV infection causes symptoms including leaf mosaic, chlorosis, deformation, necrosis, stunting, and even plant death in *Brassica* crops (Walsh *et al.* 2002). However, TuMV control is difficult due to a wide range of host and non-persistent mode of transmission by aphids (Hughes *et al.* 2002). Therefore, improvement of genetic-based TuMV resistance is one of viable and effective crop protection strategies.

To date, multiple sources of TuMV resistant genes or quantitative trait loci (QTLs) have been identified in *Brassica* species. In Chinese cabbage, QTLs *Tu1* and *Tu2* are associated with a dominant resistance to TuMV(C4) at the seedling stage (Zhang *et al.* 2008), while the other dominant QTLs, *TuR1* and *TuR2*, are resistance to TuMV(C3) (Cui *et al.* 2008). *ConTR01*, *TuRBCH01*, *TuRB07*, *TuRB01b*, and *TuRBCS01* were reported to be dominant genes found in *B. rapa* (Jin *et al.* 2014, Li *et al.* 2015, Lydiate *et al.* 2014, Rusholme *et al.* 2007, Xinhua *et al.* 2011). Additionally, several recessive genes including *retr01*, *retr02*, and *trs* expressing a broad-spectrum resistance (BSR) to TuMV were also identified (Kim *et al.* 2013, Nellist *et al.* 2014, Qian *et al.* 2013, Rusholme *et al.* 2007). However, resistance mediated by dominant genes is limited by the specialization of pathogens and is vulnerable to a single loss-of-function mutation in the virus’s avirulence gene (Li *et al.* 2020). While resistance mediated by recessive genes demonstrates relatively greater stability against viral BSR, this is due to mutations in genes that encode host factors critical to viral infection (Hashimoto *et al.* 2016). As viruses lack metabolic machinery, they hijack host translation initiation factors to replicate during infection (Imai *et al.* 2023). The viral protein genome-linked (VPg) of TuMV functions similarly to the cap structure and has shown to bind to host plant translation initiation factors (Wang *et al.* 2022). The eukaryotic initiation factor 4E (eIF4E) and 4G (eIF4G) have isoforms, eIFiso4Es and eIFiso4Gs, with gene mutations associated with resistance towards potyviruses including TuMV (Robaglia and Caranta 2006, Sanfaçon 2015, Wang and Krishnaswamy 2012). Both *eIF4E.a* and *eIF4E.c* are copies of *eIF4E*, and both *eIFiso4E.a* and *eIFiso4E.c* are copies of *eIFiso4E* in *Brassica rapa*. The recessive genes, *retr01/retr02*, are recognized as defective alleles of *eIFiso4E.a*, with the *trs* locus considered to be in close proximity (Nellist *et al.* 2014, Qian *et al.* 2013, Rusholme *et al.* 2007). In parallel with genetic analyses, many TuMV resistant *Brassica* varieties/lines have been found (reviewed by Li *et al.* 2019). Despite the known resistant varieties/lines and genes within *Brassica* species, there is a need for more resistance resources and information to effectively combat the various strains/isolates of TuMV and any emerging mutants.

The seedbank at Tohoku University (Tohoku Univ. *Brassica* Seed Bank) stores around 750 germplasms (maintained with accession number) of the genus *Brassica* and related species and genera. Seeds of these Brassicaceae plants were collected through expeditions to the Mediterranean area and seed exchanges with universities and institutes across many countries, a project spanning over 50 years since Prof. Mizushima first initiated it (Tsunoda *et al.* 1980). This seed bank contains more than 90 accessions of *Brassica rapa* from Japan, UK, USA, Canada, China, Thailand, New Zealand, Egypt, Spain, Korea, India, Sweden, Pakistan, and Turkey, including not only cultivated but also wild type accessions. For the different degrees of resistance to TuMV among the accessions within this *B. rapa* collection, we conducted inoculation tests of 95 *B. rapa* accessions against 11 TuMV isolates, grouped to three clusters based on genetic background, and evaluated the degree of resistance based on disease symptom patterns and detection patterns of coat protein (CP). This analysis identified two accessions in which no virus was detected in inoculated and upper leaves against all 11 isolates. Furthermore, examination of the *eIF4E*s gene sequences in the two resistant accessions, compared to the susceptible accessions, identified several mutations with the implications towards TuMV-BSR discussed.

## Materials and Methods

### Plants and viruses

A total of 95 accessions of *Brassica rapa*, including 92 accessions from the Tohoku *Brassica* Seed Bank (URL: https://www.agri.tohoku.ac.jp/pbreed/Seed_Stock_DB/SeedStock-top.html) and three commercial cultivars, ‘Gokurakuten’ (TAKII & CO., LTD., Japan), ‘CR-seiga 65’ (Ishii Seed Grwowers CO., LTD., Japan), and ‘Harusakari’ (Watanabe Seed CO., LTD., Japan) were used in this study (Supplemental Table 1). These plants were grown under a 12 h light (14,000 lux)/12 h dark photoperiod at 23°C in a growth chamber (NK system, Osaka, Japan). Eleven isolates of Turnip mosaic virus (TuMV) were obtained from NARO Genebank and were maintained on the leaves of *Nicotiana benthemiana* (Supplemental Table 2).

### Virus inoculation and detection

Virus-infected leaves of *N. benthemiana* were homogenized in 10 x volume of 0.1 M phosphate-buffered saline (pH 8.0) on ice. These homogenates were used to mechanically inoculate new fully expanded leaves of 2–3 true leaf stage plants with the tip of a cotton swab and carborundum as previously described (Bradford 1976). Virus inoculated leaves and non-inoculated upper leaves were collected 10- and 20-days post inoculation (dpi), respectively. Immunological virus detection by western blot analysis was performed according to standard protocol (Sambrook and Russell 2001a) using antibodies to the coat protein of TuMV and CMV (Bioreba AG, Reinach, Switzerland) (Takahashi *et al.* 2018, Tian *et al.* 2020).

### Detection of cell death

Cell death in virus inoculated plants was visualized with trypan blue staining according to standard protocol (van Wees 2008). Leaves were stained by boiling for 15 min in alcoholic lactophenol [99.5% ethanol:phenol:glycerol acid 4:1:1:1 (v:v:v:v)] containing 0.1 mg/mL trypan blue. Stained leaves were decolorized in a 2.5 g/mL chloral hydrate solution overnight, then placed in 70% ethanol for imaging. Trypan blue staining is useful for qualitative cell death detection but does have limitations for quantitative cell death evaluation.

### DNA and RNA extraction and cDNA synthesis

Genomic DNA was extracted from *B. rapa* leaves by CTAB according to standard protocol (Sambrook and Russell 2001b). Total RNA was extracted from *B. rapa* leaves and TuMV-infected *N. benthemiana* leaves using TRIzol reagent (Wako Co., Japan) and kept at –80°C. Extracted total RNA were used for cDNA synthesis using a PrimeScript RT reagent kit (TaKaRa Bio Inc., Shiga, Japan) according to standard protocol (Sambrook and Russell 2001b).

### Generation and Sequencing of cDNA of eIF4E (eIFiso4E) and VPg and P1 of TuMV

The full-length cDNA of *eIF4E.a*, *eIF4E.c*, *eIFiso4E.a*, and *eIFiso4E.c* of *B. rapa* and the TuMV VPg and P1 coding sequence were generated by PCR as follows. Each 20 μL reaction mixture containing 1 μL cDNA as a template with forward and reverse primers (0.25 μM each), was prepared using the KOD-Plus polymerase (TOYOBO, Japan) according to the manufacturer’s instructions. PCR primers were designed using Geneious Prime (version 2022.2.2) as shown in Supplemental Tables 3 and 4. PCR products were gel-purified using NucleoSpin Gel and PCR Clean-up (TaKaRa Bio Inc.) following the manufacturer’s protocol. Purified DNA fragments were sequenced using the Sanger method at Eurofins Genomics (Tokyo, Japan). DNA and amino acid sequences were analyzed with Geneious Prime. The three-dimensional structure of eIF4E/eIFiso4E protein was predicated by ColabFold (version 1.5.5; viewed at https://www.rcsb.org) (Jumper *et al.* 2021, Mirdita *et al.* 2022). The identified sequences of VPg and P1 region of TuMV were submitted to NCBI with accession numbers list in Supplemental Table 5.

### Development of dCAPS markers

dCAPS mismatch primers were designed using dCAPS Finder2.0 (http://helix.wustl.edu/dcaps/) (Neff *et al.* 2002) and the corresponding reverse primers generated by Geneious Prime as shown in Supplemental Table 6. PCR was performed using 20 μL reaction mixtures containing 1 μL cDNA, 0.25 μM each of forward and reverse primers, 0.2 mM dNTPs, 1.5 mM MgSO_4_, 0.4 U of KOD-Plus-Neo (TOYOBO, Japan), and 1 x PCR buffer. PCR was performed using the following program: 94°C for 1 min, followed by 35 cycles of 94°C for 30 s, 55°C for 30 s and 72°C for 12 s according to the manufacturer’s instructions. The restriction enzyme digestions for each dCAPS marker were performed with 10 μL reaction mixtures containing 1 x buffer, 4 μL PCR product and 0.2 μL enzyme using the corresponding reaction conditions pursuant to the instructions (Takara Bio Inc.) (Supplemental Table 6). The association between each SNP and phenotypic repones to TuMV was characterized by Chi square test with jamovi (version 2.3).

### Recombination analysis and phylogenetic analysis

The P1 and CP (coat protein) sequences of eleven tested TuMV isolates and other 52 TuMV isolates obtained from the GenBank Database were subjected to recombination analysis using the programs RDP (Martin and Rybicki 2000), GENECONV (Ohshima *et al.* 2021), BootScan (Martin *et al.* 2005), MaxChi (Smith 1992), Chimaera (Posada and Crandall 2001), SiScan (Gibbs *et al.* 2000), 3Seq (Lam *et al.* 2018), LARD (Holmes *et al.* 1999), and Phylpro (Holmes *et al.* 1999), implemented in the RDP5 package (Martin *et al.* 2021). Sequences were analyzed using the default settings of each detection program and a Bonferroni-corrected *P*-value cut-off of 0.05. The RDP, BootScan, and SiScan programs used phylogenetic methods, whereas GENECONV, MaxChi, and Chimaera programs used substitution methods, while the Phylpro program used a distance comparison method. Sequences with recombination supported by at least three programs or two methods and a *P*-value of <10^–6^ were considered as ‘clear’ recombinants; otherwise, they were noted as ‘tentative’ recombinants (Li *et al.* 2017). The phylogenetic tree was constructed using the Maximum Likelihood (ML) method with the genetic distance model Jukes-Cantor after cluster omega alignment in MEGA7 (Kumar *et al.* 2016). Data sets were bootstrapped (1,000 replicates) to assess phylogenetic tree confidence values, with bootstrap values <50% omitted. Fifty-two reported TuMV isolates were used as references for TuMV phylogenetic groups (Kawakubo *et al.* 2021, Yasaka *et al.* 2017). In addition, one narcissus late season yellows virus (NLSYV) (NC_023628) was used as the outgroup.

## Results

### Molecular phylogenic analysis of Japanese TuMV isolates

The CP region sequences of 11 TuMV isolates were obtained from the NARO Genebank. The sequences showed 87.5%–99.8% and 92.7%–100% identities at nucleotide (nt) and amino acid (aa) levels with other 52 reference TuMV sequences, respectively. Numerous studies have indicated that among the polyproteins encoded by TuMV, the P1 protein is the most variable potyviral protein and also involved in the adaptive process and host range specificity (Nigam *et al.* 2019). The interaction between VPg protein and eIF4E or eIFiso4E of *Brassica* determined the virulence (Nellist *et al.* 2014). Therefore, the sequences of the P1 and VPg region of eleven TuMV isolates were also determined. The P1 region sequences shared identities of 77.9%–100% and 75.7%–100% at nt and aa levels with reference sequences, respectively. The VPg region sequences shared identities of 79%–100% and 88.5%–100% at nt and aa levels with reference sequences, respectively. Recombination events were surveyed among the CP, P1 and VPg regions of TuMV (see Materials and Methods). No ‘clear’ recombination was detected in the CP and VPg region of 11 TuMV isolates. On the other hand, four ‘clear’ recombination events were found in the P1 gene (Supplemental Table 7). TuMV isolates (260137, 715062, 260135, 260134) were recombined with one world-B isolate and one Asian-BR isolate. CP, VPg and P1 sequences of TuMV isolates in which recombination was not detected were used in the phylogenetic analysis. The eleven TuMV isolates were clustered into three groups corresponding to Basal-B, Asian-BR, and World-B (Supplemental Fig. 1A) according to the TuMV isolate CP sequences. TuMV (260228) was clustered into the Basal-BR group, while TuMV (715062, 260135, 260137, 260134) fell into the Asian-BR group, then TuMV (104047, 260136, 260133, 715054, 715066, 715027) allocated to the World-B group (Supplemental Fig. 1A). The eleven TuMV isolates with VPg sequences were clustered into two groups, Asian-BR and World-B. The seven TuMV isolates without P1 sequence recombination were clustered into two groups (Basal-B and World-B) (Supplemental Fig. 1B). According to the phylogenic and recombination analysis results above, five TuMV clusters were identified for the eleven TuMV isolates (Supplemental Table 8).

### Response of 95 *B. rapa* accessions to TuMV (260135)

**Fig. 1.**
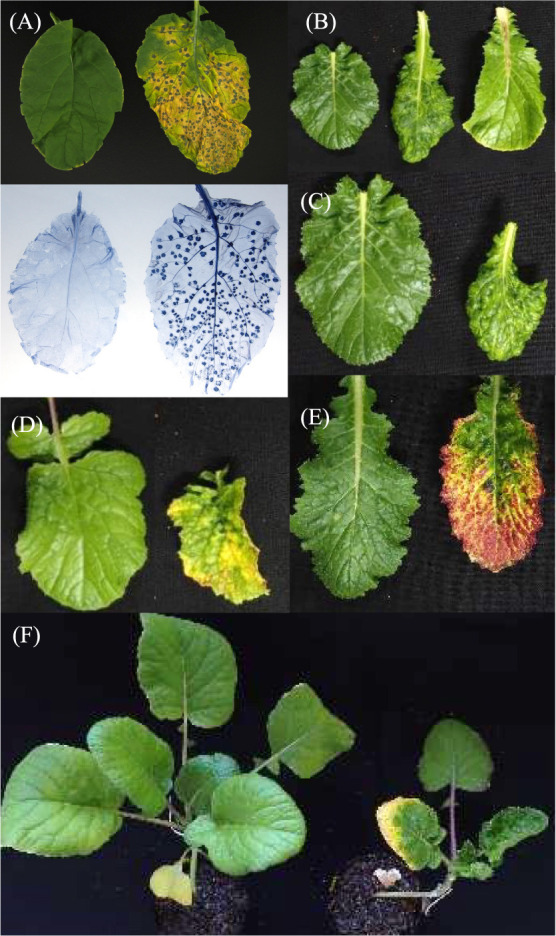
Typical symptoms developed in TuMV infected *B. rapa*. The symptoms were identified visually up to 20 days post inoculation. Necrosis was visually identified then detected by trypan blue staining in *B. rapa* accessions. A healthy and necrotic leaf (A), a healthy, mosaic and petiole necrotic (B), a healthy and deformed leaf (C), a healthy and chlorotic leaf (D), a healthy and red leaf (E), and a healthy and stunted plant (F).

**Fig. 2.**
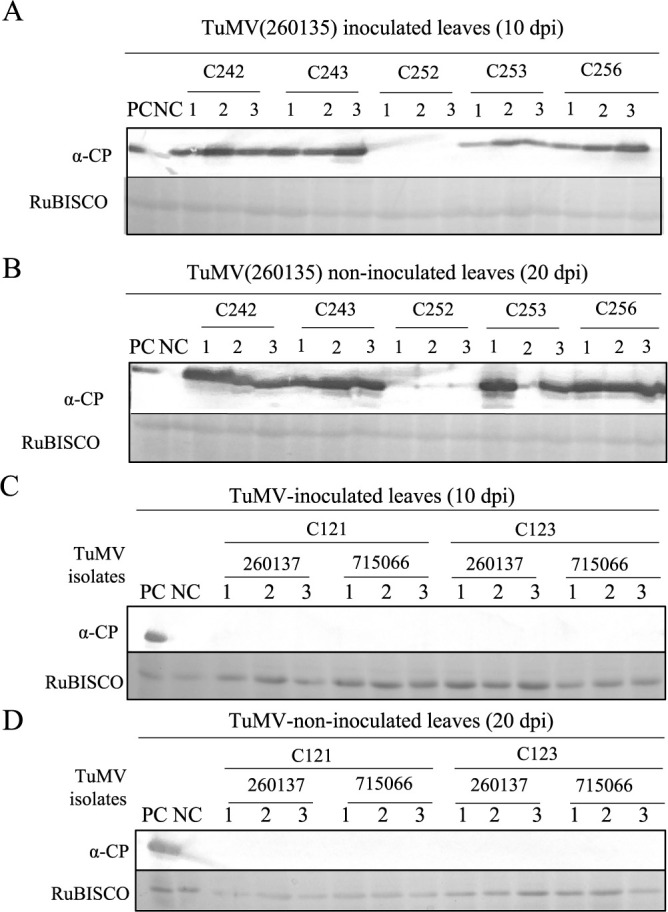
Detection of TuMV coat protein in different *B. rapa* accessions. TuMV coat protein accumulation detected in TuMV inoculated leaves at 10 dpi (A, C), TuMV non-inoculated upper leaves at 20 dpi (B, D), by western blot analysis using antibody to coat protein and Rubisco as control. PC and NC represent positive and negative controls. C252, no-symptoms and no-infection (no-CP), C256, no-symptoms but infected (CP detected), C242, C243 and C253, with symptoms and infection (see Table 1); C121 and C123, no-symptoms and no-infection (no-CP) (see Table 2).

Most of TuMV isolates belonging to Asian-BR group were from East Asian and could infect both of *B. rapa* and *Raphanus* species, resulting in a massive crop loss (Ohshima 2013). It is considered to be potentially high-risk group that could cause significant damage in Japan. Based on the CP and VPg sequences, TuMV (260135) were grouped in Asian-BR group and, as a first step, we used this isolate as a representative. To initially assess responses to TuMV in *B. rapa* accessions from Tohoku Univ. *Brassica* Seed Bank, 95 *B. rapa* accessions with 3 replications were sap-inoculated with TuMV (260135). Seventy-three *B. rapa* accessions exhibited symptoms of TuMV including systemic chlorosis, systemic necrosis, systemic mosaic, leaf deformation, red leaves, and stunting (Fig. 1, Table 1). Fourteen accessions, C121, C123, C146, C155, C252, C256, C336, C339, C430, C471, C482, C483, ‘Gokurakuten’, and ‘Harusakari’, did not exhibit symptoms up to 20 dpi. All susceptible accessions and, despite the lack of symptoms (C256, C336, C339, C430, ‘Gokurakuten’, and ‘Harusakari’) were systemically infected with the TuMV isolate, confirmed by western blot analysis (Fig. 2, results for some accessions are shown, Table 1). Western blot analysis of the remaining seven accessions, C121, C123, C146, C155, C252, C471, and C483, observed no detectable accumulation of TuMV-CP in inoculated and non-inoculated upper leaves (Fig. 2, Table 1), suggesting that TuMV (260135) is unable to proliferate in these accessions. Thus, the seven accessions are regarded to have an extreme resistance (ER) trait to TuMV (260135) (Table 1). One to two of three tested plants in the five accessions, C149, C464, C465, C466 and C473, showed no symptom (NS) and non-accumulation of the coat protein (Table 1), suggesting that the extreme resistance trait was segregated in the populations. The thirteen accessions without symptoms up to 20 dpi, were collected from USA, Canada, Egypt, New Zealand, Spain, and Japan, including two Japanese turnip cultivars, ‘Hijiori-kabu’ and ‘Akane-kabu’ (Table 2). Next, we investigated the responses of these thirteen accessions to the remaining ten isolates from the three TuMV clusters.

### Response of thirteen *B. rapa* accessions to three TuMV clusters

**Fig. 3.**
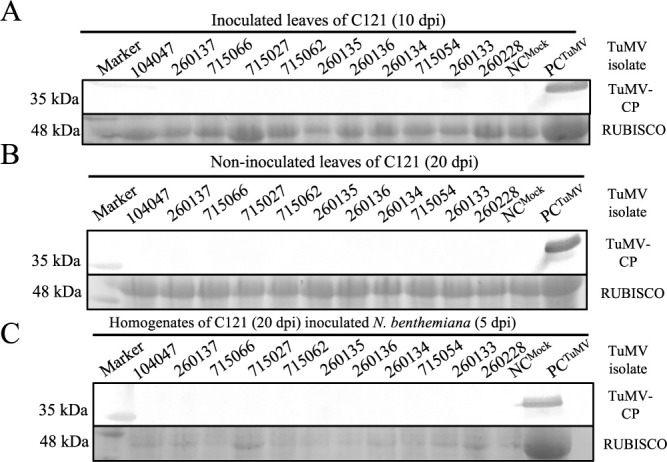
Detection of TuMV coat protein in C121. C121 was inoculated with eleven TuMV isolates, the homogenates of TuMV inoculated C121 leaves was used to inoculate *Nicotiana benthemiana*. TuMV coat protein accumulation was evaluated in TuMV inoculated leaves and non-inoculated leaves of C121 at 10 dpi (A), 20 dpi (B), and homogenates of C121 inoculated *N. benthemiana* at 5 dpi (C) by western blotting using TuMV coat protein antibody. TuMV coat protein was undetectable in inoculated and non-inoculated leaves of C121, and homogenates of C121 inoculated *N. benthemiana* leaves.

The thirteen TuMV (260135)-resistant *B. rapa* accessions were examined for their responses to ten other TuMV isolates. Phenotypic responses were identified by combing TuMV accumulation amounts with symptom development in inoculated and non-inoculated upper leaves (Fig. 2C, 2D, Table 2). In two USA accessions (C121 and C123), despite the appearance of stunted growth and mosaic on leaves against some TuMV isolates (Supplemental Fig. 2), both accessions were free of leaf necrosis, and no virus CP accumulation was detected throughout the experimental period (20 dpi) (Fig. 3A, 3B). Furthermore, the homogenates of TuMV inoculated leaves at 20 dpi was used to inoculate *N. benthemiana*, but no TuMV-CP was detected in the subsequently inoculated leaves at 5 dpi (Fig. 3C). Interestingly, one Spanish wild turnip (C471) exhibited ER to most TuMV isolates, but not TuMV (260133). Six accessions (C121, C123, C252, C466, C471, and C483) exhibited ER to more than five TuMV isolates (Table 2).

Various phenotypic responses were induced in the thirteen *B. rapa* accessions by isolates from the five TuMV clusters (Table 2). TuMV (260228), a cluster 3 isolate from genus *Leucocoryne*, could only infect ‘Hijiori-kabu’ (C473). These results indicated that the resistance responses induced between the thirteen accessions and three TuMV clusters may be mediated by different genetic factors between plant and virus. Most notably, C121 and C123, exhibiting ER against three TuMV clusters, may contain genetic factors responsible for a broad-spectrum resistance (BSR) to TuMV (Fig. 3).

### Polymorphisms in eIF4E and eIFiso4E in *B. rapa* accessions

**Fig. 4.**
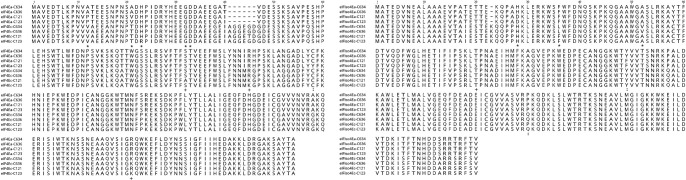
Amino acid polymorphisms in *eIF4Es* and *eIFiso4Es* among TuMV resistant accessions (C121 and C123) and TuMV susceptible accessions (C634 and C636) of *B. rapa*. ‘*’ represents amino acid substitutions located at neither dorsal surface nor cap-binding site of eIF4E and eIFiso4E, ‘+’ represents amino acid substitutions located at dorsal surface of eIF4E and eIFiso4E, ‘:’ represents amino acid substitutions located at cap-binding site of eIF4E and eIFiso4E.

Plant eukaryotic initiation factors, eIF4E and eIFiso4E, can interact with the VPg of potyviruses, necessary for viral propagation in plant cells. The *eIFiso4E* gene in *B. rapa* is strongly linked to the *Brassica* recessive resistance genes *retr01/retr02* and *trs* (Kim *et al.* 2013, Nellist *et al.* 2014, Qian *et al.* 2013) and dominant resistance gene, ConTR01 (Rusholme *et al.* 2007). In those studies, significant polymorphisms in eIF4E and eIFiso4E were detected between resistant and susceptible varieties, respectively. To search the significant polymorphisms between resistant and susceptible lines used in the present study, we first determined the cDNA sequences of *eIF4E* and *eIFiso4E* of resistant (C121 and C123) and susceptible (C634 and C636) accessions. Based on sequencing analysis, copies of *eIF4E* and *eIFiso4E* show strong similarity. However, four amino acid substitutions were located at the dorsal surface of eIF4E and eIFiso4E, while three amino acid substitutions were identified at the cap-binding site of eIF4E and eIFiso4E, according to 3D protein structure predictions using Colabfold2 (Fig. 4, Supplemental Fig. 3). In eIF4E.a, the 21^st^ amino acid residue is V in accessions C121 and C123, which differs from the A found at the same position (A21) in accessions C634 and C636. In eIFiso4E.a, H27 (28^th^ in eIFiso4E.c), in C634 and C636 differs from the D27 in C121 and C123 (H27D). However, another eIFiso4E.a polymorphism was identified as S79 in C123, and T79 in C121, C634 and C636. Also, as reported in Rusholme *et al.* (2007), two polymorphisms for eIFiso4E.c, F36L and V52A, were found. Finally, Q150 was observed for eIFiso4E.c in susceptible accessions and C123, which differed from P150 in C121.

The 36^th^ amino acid in eIFiso4E.c has been reported as important in protein structure stabilization (Li *et al.* 2018). Therefore, we designed a dCAPS marker at this substitution site and then conducted genotyping for the remaining 91 accessions. For eIFiso4E.c, L36 was identified in 25 susceptible accessions, while F36 was observed in the others. Through a sequencing analysis of other eleven accessions, we observed 42 nucleotide substitutions containing sixteen non-synonymous substitutions (Supplemental Table 9). Thirteen dCAPS markers were designed for the non-synonymous substitutions and then the genotyping was conducted. The genotypes for the 95 *B. rapa* accessions were identified and are shown in Supplemental Table 10. Chi-square analysis indicated strong association with TuMV resistance/susceptibility for five amino acid substitutions, P12A and V21A in eIF4E.a, A35V in eIF4E.c, and F36L and Q150P in eIFiso4E.c. (Table 3).

### Co-infection with TuMV and CMV

**Fig. 5.**
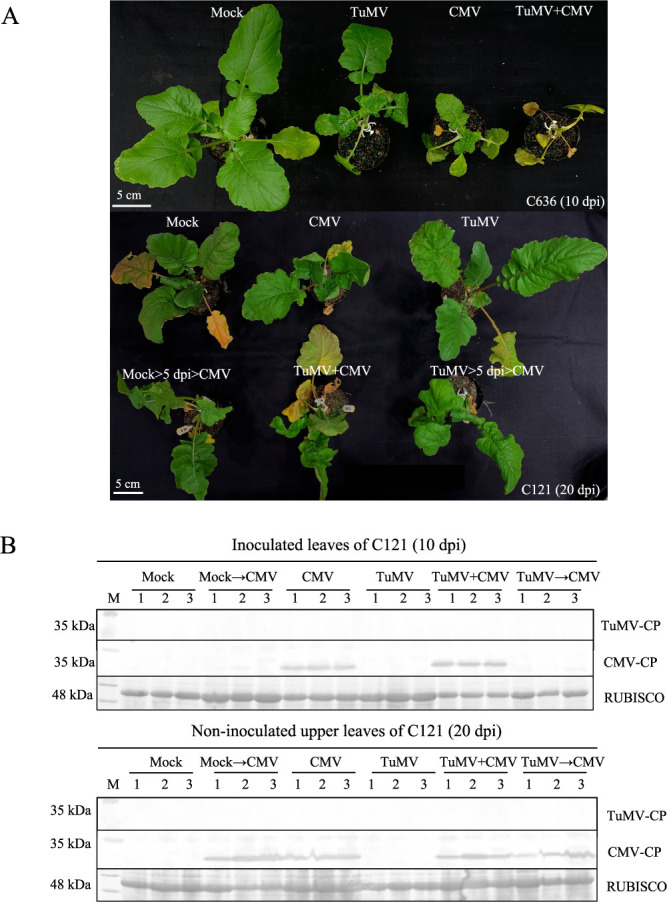
Detection of coat protein of TuMV and CMV in C121 and C636 co-infected with TuMV and CMV. The responses to TuMV, CMV, coinfection of TuMV and CMV inoculation of C636 and C121 plants were characterized. Symptom development was observed at 20 dpi, CMV infection induces stunting in C121 and C636, and co-infection induced plant death of C636 at 20 dpi (A), no TuMV CP accumulation was detected in C121, whereas CMV CP was detected in C121 from both inoculated and non-inoculated upper leaves (B).

In nature, plants are forced to face diverse pathogens, and co-infection is common. Co-infection tends to alter disease severer compared to single inoculation (Fang *et al.* 2021, Kudela *et al.* 2010, Moura *et al.* 2005). Alongside TuMV, CMV is another pathogen for field-grown *Brassica* crops, and another potential threat. TuMV and CMV are genetically different, belonging to different families. To evaluate the co-infection response towards TuMV and CMV of a TuMV-resistant *B. rapa* accession, C121 was inoculated with TuMV, CMV, TuMV+CMV, or TuMV then CMV with an interval of five days. The TuMV-susceptible accession, C636, was used for comparison. For the TuMV-susceptible accession, C636, co-inoculation with TuMV and CMV plant resulted in plant death at 10 dpi (Fig. 5A). For the TuMV-resistant accession, C121, stunting was observed for the CMV inoculated plant, whereas no symptoms were observed for the TuMV inoculated plant. Additionally, no TuMV was detected in the inoculated leaves and upper leaves, while CMV was detected from both (Fig. 5B). In the five-day interval inoculation, no CMV was detected in inoculated leaves at the fifth day after TuMV inoculation but was detected in the upper leaves on the fifteenth day (Fig. 5B). These results suggest that TuMV and CMV co-infection neither disrupts the TuMV resistance of C121 nor decreases CMV susceptibility.

## Discussion

*Brassica* crops are economically important, providing edible roots, leaves, stems, buds, flowers, and seeds. TuMV causes diseases in field-grown *Brassica* crops, decreasing production by more than 30% (Lv *et al.* 2020). Screening for resistant accessions in *Brassica* is important to breed TuMV resistant *Brassica* crops. In this study, 92 *B. rapa* accessions were employed from the Tohoku Univ. *Brassica* Seed Bank alongside three commercial *B. rapa* cultivars to identify new TuMV resistance resources. Through a survey of responses to TuMV (260135) in 95 *B. rapa* accessions, diverse phenotypic responses were identified. For example, systemic virus spread was observed in most *B. rapa* accessions accompanied by various symptoms including systemic mosaic, leaf deformation, and stunting. These responses reflect the genetic variability of *B. rapa* genetic resources. On the other hand, 13 out of 95 accessions showed extreme resistance, with no detected TuMV (260135) replication. They were collected from USA, Canada, Spain, and Japan. Also, six accessions, C121 and C123 (USA), C252 (Japan), C466 (Egypt), C471 (Spain), and C482 (Japan), exhibited resistance to more than five different TuMV isolates. Moreover, two USA accessions (C121 and C123) exhibited extreme resistance to all eleven examined TuMV isolates without CP detection, although the appearance like overly activated ETI (mentioned later) was observed against two TuMV isolates. Eventually, two *B. rapa* accessions were identified from the genetic resources collected from abroad showing broad-spectrum resistance (BSR) to Japanese TuMV isolates. Their resistance to overseas TuMV is unclear, and further inoculation tests with additional TuMV isolates are needed to fully evaluate BSR with respect to TuMV. However, the accessions used in this study showed resistance to several isolates belonging to cluster 1 to cluster 5. TuMV isolates in these clusters may come from the Asian-BR, Basal-B and World-B groups, distributed in Middle East Asia, East Asia, and Southeast Asia (Kawakubo *et al.* 2021). Therefore, accessions C121 and C123 are excellent candidates to develop TuMV-resistant varieties. Currently, *B. rapa* TuMV resistance resources are primarily reported in Chinese cabbage lines. In line RLR22, derived from line BP079, BSR against TuMV mediated by *ConTR01* and *retr01* was identified (Rusholme *et al.* 2007). In a separate finding, a different TuMV BSR was observed in line BP8407 to be mediated by a single recessive gene, *retr02* (Qian *et al.* 2013). Additionally, in line SB18 and SB22, another BSR mediating recessive gene, *trs*, was identified. It was considered as an allele of *retr01/retr02* or a gene closely linked to *retr01/retr02* (Kim *et al.* 2013). An HR-mediated single dominant resistance gene, *BcTuR3*, was found in the line Duanbaigeng (Ma *et al.* 2010), and another single dominant resistance gene, *TuRBCS01*, also identified in line 8407 (Li *et al.* 2015). As well as the Chinese cabbage lines, a single dominant resistance, *TuRBCH01*, was also mapped in pak choy (Xinhua *et al.* 2011), with resistance to TuMV observed in the turnip cultivar UG1 (Shattuck 1992). Despite the existing range of identified resistance genes in *B. rapa*, additional resistance resources are preferable. For the growing threat of unexpected foreign viral incursion, both C121 and C123 lines may serve as a breeding resource to develop TuMV resistant varieties.

Studies on interactions between *B. rapa* and TuMV are focused on a “one to one” binary plant-microbe interaction. However, in nature, field-grown *Brassica* crops are faced with a range of pathogens and environmental conditions. Therefore, the practical impact of viral co-infection from pathogens with distinct genetic backgrounds requires consideration for resistance breeding in *Brassica* crops. The USA accession, C121, was immune to TuMV, but was susceptible to CMV. Also, the TuMV susceptible accession, C636, was also infected by CMV. Additionally, co-infection of TuMV and CMV was observed to promote disease development resulting in death for C636. However, co-infection did not mitigate TuMV resistance in C121. Based on the above findings, our study suggests that C121 (and likely C123) might have robust broad-spectrum TuMV resistance, which differs from the resistance of lines and cultivars in previous reports (Li *et al.* 2016, Nellist *et al.* 2014, Rusholme *et al.* 2007). Both accessions may be highly effective and practical genetic resources.

Plants evolved complex immune systems to defend against diverse pathogens. The first line of defense is PAMP (Pathogen-associated molecular patterns)-triggered immunity (PTI), and the second is effector-triggered immunity (ETI). The ETI is activated by pathogen virulence factor recognition by plant effectors, such as R proteins expressed by dominant resistance genes (Jones and Dangl 2006). R gene-mediated resistance usually induces localized programmed cell death, known as the hypersensitive response (HR), to prevent infection spread. Overly activated ETI can cause stunted growth of plant (Waheed *et al.* 2021). The stunting observed in C121 against two TuMV isolates was possibly due to the overly activated ETI.

Currently, more than ten TuMV resistance genes in *Brassica* crops are identified (Lv *et al.* 2020). *TuRB01* to *TuRB05* were identified in *Brassica napus* (Hughes *et al.* 2003, Walsh *et al.* 1999). *TuRB05*-mediated resistance induces HR necrosis to limit TuMV isolate systemic spread, while *TuRB01* to *TuRB04*, identified in *B. rapa*, contribute to an extreme TuMV resistance. A similar or identical allele of *TuRB01* in *B. napus*, *TuRB01b*, mapping on *B. rapa* A6 confers extreme resistance to TuMV(UK1), and HR resistance to TuMV(GK1) (Lydiate *et al.* 2014). Furthermore, the multiple copies of eukaryotic translation initiation factors in *Brassica rapa* facilitate redundancy and influence the interactions with TuMV. It is reported that the combined action of *retr01* [*eIFiso4E.a*] and *ConTR01* [*eIFiso4E.c*] from Chinese cabbage accession BP079 contributed a broad-spectrum HR resistance to TuMV isolate CDN1 and CZE1 (Rusholme *et al.* 2007). Mis-splicing induced by G insertion in *eIFiso4E.a* of *B. rapa* contributed to the establishment of *retr02*-mediated broad-spectrum TuMV resistance (Li *et al.* 2016, Nellist *et al.* 2014). However, no such insert G was found in *eIFiso4E.a* among the *B. rapa* accessions used in this study. Our *B. rapa* accessions, C121 and C123, also had no insert G, showing extreme resistance, but not HR. Therefore, it appears that the broad-spectrum resistance of C121 and C123 is a novel TuMV resistance, regulated by a mechanism other than a *retr02*-mediated one.

Sequencing and dCAPS analysis identified four and three amino acid substitutions on the dorsal surface and cap-binding sites of the eIF4E/eIFiso4E proteins, respectively. And significant associations with TuMV resistance/susceptibility were identified for A12P and V21A in *eIF4E.a*, A35V in *eIF4E.c*, and F36L and Q150P in *eIFiso4E.c* according to chi square evaluation. Two major structural features are related to the cap-binding site and dorsal surface of eIF4E/eIFiso4E. The m7p cap and cap-like structure, such as the TuMV VPg, may intercalate with the cap-binding site of eIF4E, with eIF4G binding to the eIF4E dorsal surface, forming the complex, eIF4F, used in translation initiation (Borden 2016). It is reported that F36 in *eIFiso4E.c* is highly conserved and crucial for the allosteric regulation of the *eIFiso4E.c* protein. Thus, F36L may be critical for the interaction between the TuMV VPg and *eIFiso4E.c* protein, and TuMV infection (Li *et al.* 2018). Based on the analysis of the three-dimensional structure (Supplemental Fig. 3), it was observed that Q150P resides within the cap-binding site of eIFiso4E. Consequently, it is hypothesized that mutation at 150^th^ amino acid of eIFiso4E.c may also play a critical role in mediating the interaction between TuMV VPg and eIFiso4E.c protein, potentially influencing TuMV infection. The residues A12P, V21A, and A35V, situated neither on the dorsal surface nor in the region of the cap-binding site, present challenges in inferring their significance in the protein’s functionality. Further investigations are warranted to elucidate the potential roles of these amino acids in protein function. Additionally, some amino acid substitutions were also observed in the dorsal surface. These may inactivate interactions between eIF4G and eIF4E, resulting in decreasing the formation rate of the translation initiation complex and preventing viral proliferation. In contrast, it was observed that certain accessions harboring the aforementioned five amino acid substitutions did not demonstrate resistance to TuMV. This observation implies the involvement of additional susceptibility factors, thereby indicating a complex interplay of genetic elements influencing the resistance phenotype.

Whether the observed resistance is qualitative or quantitative is not yet determined and requires further clarification. To achieve this, a crossed population between C121 and C634 is being produced. This will contribute significantly to increasing available genetic material for TuMV resistance breeding as previously mentioned and will also contribute to TuMV resistance gene identification and elucidation of the BSR resistance mechanism. Additionally, whether C121 and C123, the two accessions showing TuMV-BSR, will demonstrate resistance to strains/isolates from other countries is important to evaluate. We would like to pursue this in collaboration with researchers world-wide to improve to TuMV-resistance breeding in *B. rapa*.

## Author Contribution Statement

AT and HK designed the research. AT performed the research, AT and HK analyzed the data. All authors discussed and wrote the manuscript.

## Supplementary Material

Supplemental Figures

Supplemental Tables

## Figures and Tables

**Table 1.** Symptoms and phenotypic responses induced by TuMV (260135) strains in 95 *Brassica rapa* accessions

Accession No.	Symptom*^a^*	Phenotypic response*^b^*		Accession No.	Symptom*^a^*	Phenotypic response*^b^*
C101	SM, LD	+		C474	ST, LD, SN	+N
C102	SM, ST	+		C475	ST, LD	+
C103	SM	+		C476	SM	+
C104	SM, LD	+		C477	SM, ST, LD, SC	+
C105	SM	+		C478	SM, LN	RN/+
C107	SM, LD	+		C479	SM(2/3); NS(1/3)	+(2/3); ER(1/3)
C108	SM, LD	+		C482	NS	R(1/3); ER(2/3)
C109	SM, ST, LD	+		C483	NS	ER
C110	SM, ST, LD	+		C503	SM, ST, LD, LN	RN/+
C112	SN, ST, SC	+N		C505	SC	+
C120	SC	+		C506	SM	+
C121	NS	ER		C507	SM, LD	+
C123	NS	ER		C508	SM, LD	+
C137	LD, ST	+		C509	SC	+
C142	SM, ST, LD	+		C560	SM, ST, LN	+
C144	SM, ST, LD, RL	+		C632	SM, ST, LN	RN/+
C145	SM, ST, LD, RL	+		C633	SM, ST	+
C146	NS	ER		C634	SM, LD	+
C147	SM, ST, LD	+		C635	SM, ST	+
C149	slightST(1/3); NS(2/3)	+(1/3); ER(2/3)		C636	SM, LD, LN, ST	RN/+
C151	SM, ST, LD	+		C637	SM, ST	+
C152	ST	+		C642	SM, ST, LD	+
C155	NS	ER		C646	SM, ST	+
C220	SM, LD	+		C647	SM, ST, LD	+
C240	SM, ST, LD	+		C648	SM, ST	+
C241	SM, ST, LD, SN	+N		C651	SM, ST	+
C242	NS(2/3); ST, LD, SM, SN(1/3)	+(2/3); +N(1/3)		C652	SM, ST, LD	+
C243	PN, RL	+N		C653	SM, ST, LD	+
C252	NS	ER		C654	SM, ST, LD	+
C253	SN(2/3); LD, SM(1/3)	+(1/3); +N(2/3)		C655	SM, ST, LD	+
C256	NS	+		C656	SM	+
C333	SM, ST, LD	+		C663	SM, ST	+
C334	ST	+		C664	SM, ST	+
C335	SM	+		C665	SM, ST	+
C336	NS	+		C666	SM, ST, SC	+
C337	SM, ST	+		C667	SM, ST	+
C338	PN, SM	+N		C668	SM, ST	+
C339	NS	+		C669	SM, ST	+
C430	NS	+		C670	SM, ST	+
C455	LD	+		C701	SM, ST, LD	+
C464	NS(2/3); LD, ST, SN(1/3)	ER(2/3); +N(1/3)		C702	SM	+
C465	NS(2/3); SM, ST(1/3)	ER(2/3); +N(1/3)		C703	SM	+
C466	NS(2/3); SM, ST(1/3)	ER(2/3); +N(1/3)		C705	SM, ST	+
C468	ST, LC, LN	RN/+		C801	SM, ST	+
C470	SM, LD	+		CR-seiga	LN	RN/+
C471	NS	ER		Gokurakuten	NS	+
C472	SM, ST, LD	+		Harusakari	NS	+
C473	SM(1/3); NS(2/3)	+(1/3); ER(2/3)				

*^a^* Symptom code: SC = systemic chlorosis, SN = systemic necrosis, SM = systemic mosaic, LN = leaf necrosis, PN = petiole necrosis, LD = leaf deformation, RL = red leaves, NS = no symptom, ST = stunting; *^b^* Phenotypic response code: ER = extreme resistance with no detectable infection, +N = systemic infection with necrosis, + = systemic infection without necrosis, RN/+ = local necrosis in inoculated leaves and systemic spread without necrosis.

**Table 2.** Phenotypic responses and symptoms induced by eleven TuMV isolates in thirteen TuMV (260135)-resistant accessions of *Brassica rapa*

Symptoms*^a^*											
	TuMV (260228)	TuMV (715062)	TuMV (260135)	TuMV (260134)	TuMV (260137)	TuMV (260136)	TuMV (715054)	TuMV (260133)	TuMV (715066)	TuMV (715027)	TuMV (104047)
C121	ST	NS	NS	NS	NS	NS	NS	NS	ST	NS	NS
C123	NS	NS	NS	NS	NS	NS	NS	NS	NS	SM	NS
C146	SM, LD	NS	NS	n/a	LN, SM	SM, ST(1/3); NS(2/3)	SM, LN	NS	SM, ST, LD	SM	SM, ST(1/3); NS(2/3)
C149	NS	SM, LD	slightST(1/3); NS(2/3)	n/a	SM, ST(1/3); NS(2/3)	SM, LD, ST(1/3); NS(2/3)	NS	SM(1/3); NS(2/3)	ST, LD(1/3); NS(2/3)	NS	NS
C155	MS	NS	NS	n/a	NS	NS(2/3); SM, ST, LD(1/3)	ST(1/3); NS(2/3)	ST(2/3); NS(1/3)	SM, ST, LD	SM, ST, LD	SM, ST, LD
C252	NS	NS	NS	NS	NS	NS	NS	NS	SM, LD	NS	SN, LD
C464	NS	SM, ST	NS(2/3); LD, ST, SN(1/3)	n/a	SM, ST	SN, LD, ST	NS	SN, LD, ST	ST, SM	ST, SM	SM, ST, LD
C465	SM	ST, SM	NS(2/3); SM, ST(1/3)	n/a	ST, SM	SM	NS	SM	NS	NS	SM
C466	NS	NS	NS(2/3); SM, ST(1/3)	n/a	NS	SM	NS	SN	SM, ST, LD	SM, ST, LD	NS
C471	NS	NS	NS	NS	NS	NS	NS	SN(1/3); NS(2/3)	NS	NS	NS
C473	NS	LN, SM	SM(1/3); NS(2/3)	n/a	SM	LD, LN, ST	NS	SM, LN	SM, ST, LN	SM	NS
C482	NS	ST, SM	NS	n/a	NS	SM	NS	SN(1/3); ST, SM(2/3)	NS	LS(1/3); NS(2/3)	NS
C483	NS	NS	NS	n/a	NS	NS	SM, ST	LN, SM, ST	NS	NS	NS
Phenotypic response*^b^*											
	TuMV (260228)	TuMV (715062)	TuMV (260135)	TuMV (260134)	TuMV (260137)	TuMV (260136)	TuMV (715054)	TuMV (260133)	TuMV (715066)	TuMV (715027)	TuMV (104047)
C121	ER	ER	ER	ER	ER	ER	ER	ER	ER	ER	ER
C123	ER	ER	ER	ER	ER	ER	ER	ER	ER	ER	ER
C146	ER	+(1/3); ER(2/3)	ER	n/a	+(2/3); ER(1/3)	+(1/3); ER(2/3)	+	+(2/3); ER(1/3)	ER	+	+(1/3); ER(2/3)
C149	ER	+	+(1/3); ER(2/3)	n/a	+(1/3); ER(2/3)	+(1/3); ER(2/3)	ER	+(1/3); ER(2/3)	+(1/3); ER(2/3)	+	+(1/3); ER(2/3)
C155	ER	ER	ER	n/a	ER	+(1/3); ER(2/3)	+(1/3); ER(2/3)	+(2/3); ER(1/3)	+	+	+
C252	ER	ER	ER	ER	ER	ER	ER	ER	+(1/3); ER(2/3)	+(2/3); ER(1/3)	+
C464	ER	+(2/3); ER(1/3)	ER(2/3); +N(1/3)	n/a	+(1/3); ER(2/3)	RN/+	ER	RN/+	+	+	ER
C465	ER	+(2/3); ER(1/3)	ER(2/3); +N(1/3)	n/a	+(2/3); ER(1/3)	R(2/3); ER(1/3)	ER(2/3); +N(1/3)	+(2/3); ER(1/3)	ER	ER	ER
C466	ER	ER	ER(2/3); +N(1/3)	n/a	ER	+(1/3); ER(2/3)	ER(2/3); +N(1/3)	ER	+	+	ER
C471	ER	ER	ER	ER	ER	ER	ER	+(1/3); ER(2/3)	ER	ER	ER
C473	+(2/3); ER(1/3)	+	+(1/3); ER(2/3)	n/a	+	RN/+	ER	RN/+	+	+	ER
C482	ER	ER	R(1/3); ER(2/3)	n/a	ER	ER	R(1/3); ER(2/3)	ER(2/3); +N(1/3)	ER	+(1/3); ER(2/3)	ER
C483	R	+(2/3); ER(1/3)	ER	n/a	ER	ER	R	+N	ER	+(2/ 3); ER(1/3)	+(2/3); ER(1/3)

*^a^* Symptom code: SM = systemic mosaic, NS = symptomless, ST = stunting, n/a = not applicable; *^b^* Phenotypic response code: ER = extreme resistance with no detectable infection, RN/+ = local necrosis in inoculated leaves and systemic spread without necrosis, +N = systemic infection with necrosis, + = systemic infection without necrosis, n/a = not applicable.

**Table 3.** Correlation analysis of amino acid substitution with TuMV resistance traits

Gene/ Position	Amino acid	Number of samples			Chi-square test	
		Resistance	Susceptibility		χ^2^	*P*
eIF4E.a/12	P	11	38		6.71	0.035*
	P/A	0	7			
	A	22	37			
eIF4E.a/21	V	11	34		8.55	0.014*
	V/A	0	10			
	A	2	38			
eIF4E.a/40	I	11	59		0.928	0.335
	T	2	23			
eIF4E.a/112	C	3	26		0.394	0.530
	Y	10	56			
eIFiso4E.a/108	F	7	41		1.33	0.513
	F/Y	4	17			
	Y	2	24			
eIF4E.c/35	A	9	25		8.51	0.014*
	V	1	36			
	A/V	3	21			
eIF4E.c/45	G	6	22		2.62	0.270
	G/T	4	24			
	T	3	36			
eIF4E.c/105	K	12	63		1.64	0.440
	K/R	0	1			
	R	1	18			
eIF4E.c/201	K	9	75		5.8	0.055
	K/R	3	6			
	R	1	1			
eIFiso4E.c/36	F	13	57		5.38	0.020*
	L	0	25			
eIFiso4E.c/52	A	13	58		5.09	0.078
	A/V	0	3			
	V	0	21			
eIFiso4E.c/80	I	1	11		2.57	0.277
	I/T	1	20			
	T	11	51			
eIFiso4E.c/150	P	4	37		8.23	0.016*
	P/Q	6	11			
	Q	3	34			

* denotes significant correlation (*P* < 0.05).
