# Supplementary material for: Screening Brassica rapa for broad-spectrum resistance to Turnip mosaic virus
Source: Breed Sci. 2024 Aug 27;74(4):354–65. doi: 10.1270/jsbbs.24015 (PMC11769589; doi:10.1270/jsbbs.24015)
Supplement: Supplementary file 1 — Supplemental Figures [file 74_354_s1.pdf]

Tian et al./Breeding Science
doi: 10.1270/jsbbs.24015

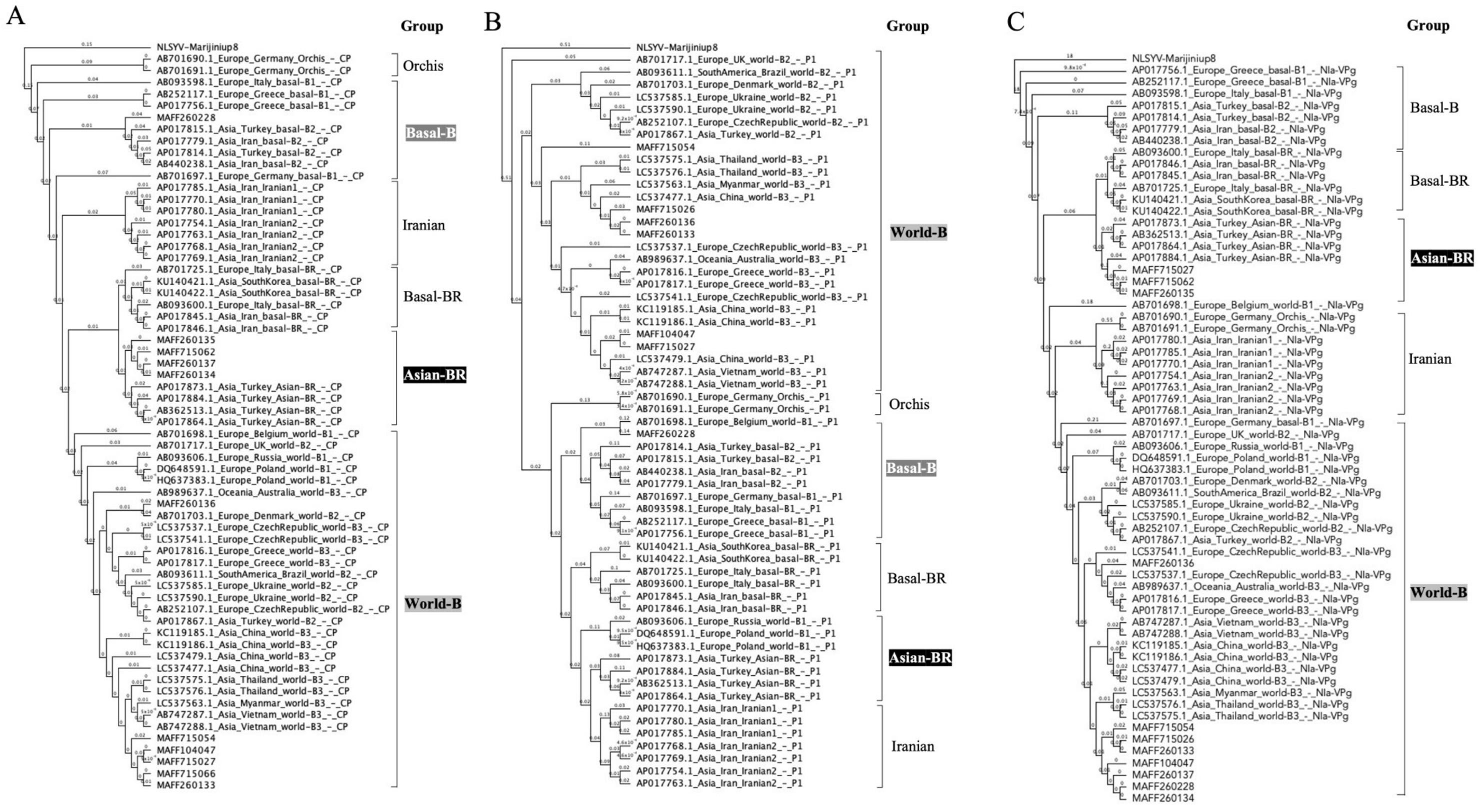

Supplemental Fig. 1 Maximum-likelihood tree of turnip mosaic virus isolates, based on eleven nonrecombinant sequences of CP and VPg region of TuMV (A, C) and seven nonrecombinant sequences of P1 region of TuMV (B) and , respectively. 52 TuMV isolates were used as reference isolates (Yasaka et al. 2017, Kawakubo et al. 2021), and they were named in the order of NCBI accession number, collection area, phylogenic group, and region of TuMV. The genomic sequences of NLSYV was used as outgroup taxa. Numbers at internal nodes indicate substitutions per site.

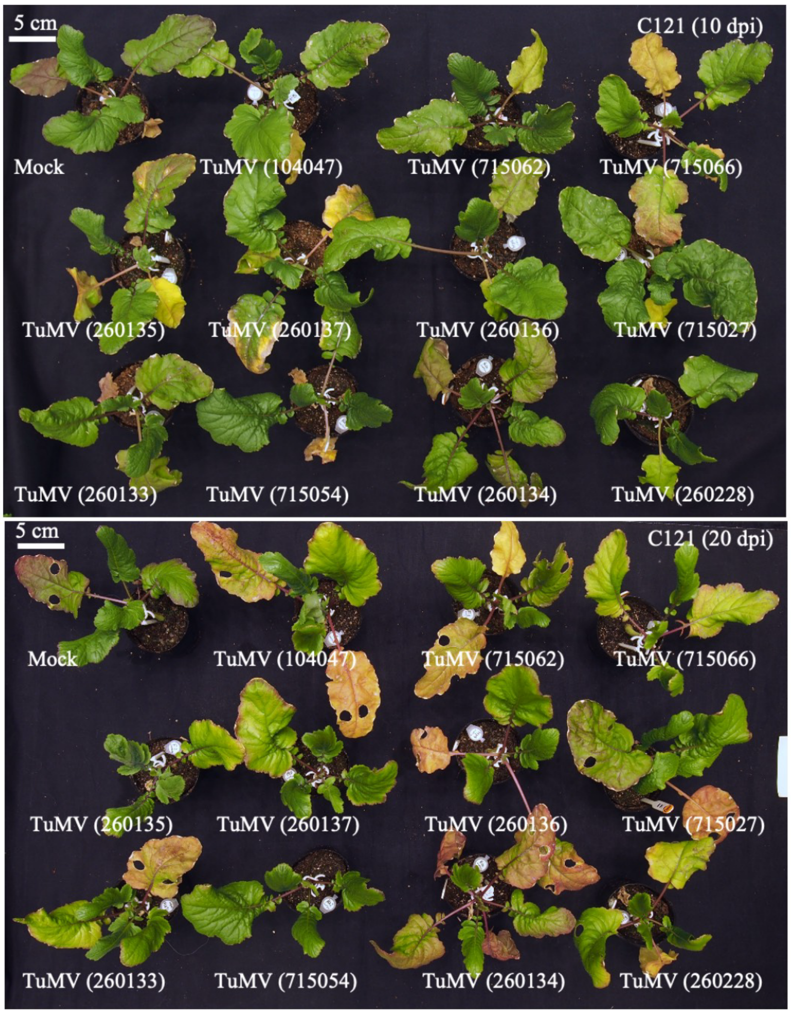

Supplemental Fig. 2 C121 was inoculated with eleven TuMV isolates, and growth stunting was observed for TuMV (260228) and TuMV (715066) inoculated plants.

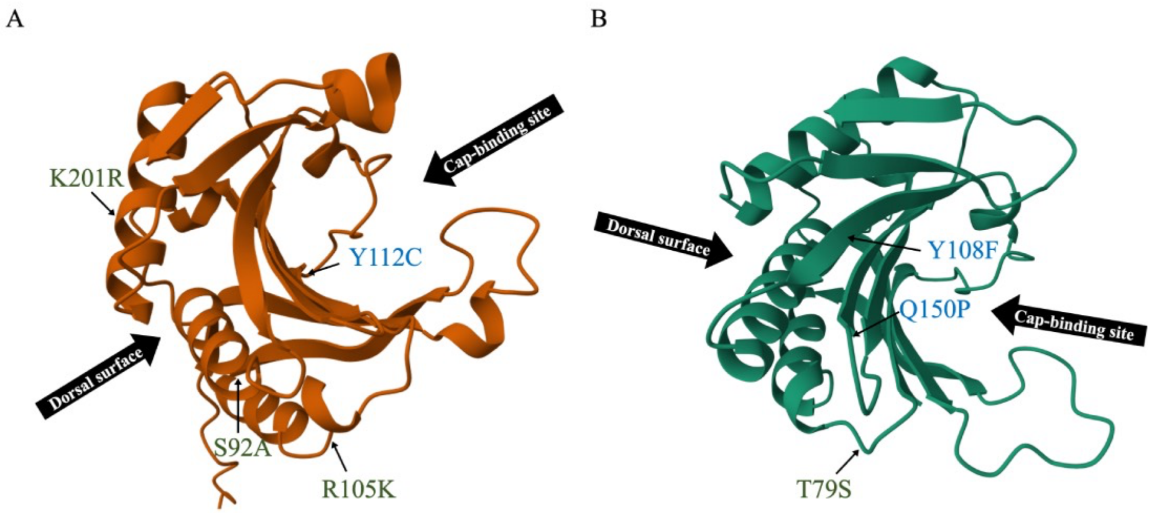

Supplement Fig. 3 A 3D structure of eIF4E.a (A)/eIFiso4E.a (B) was modelled using the eIF4E.a/eIFiso4E.a sequence in C121 by Colabfold2 and viewed at https://www.rcsb.org. eIF4E/eIFiso4E contains two major structural features, the cap-binding site and dorsal surface. The amino acid substitutions of eIF4E/eIFiso4E among TuMV resistant cultivars (C121and C123) and TuMV susceptible cultivars (C634 and C636) (Fig. 4) located at the cap-binding site and dorsal surface of eIF4E/eIFiso4E are labelled.
