# Supplementary material for: Screening Brassica rapa for broad-spectrum resistance to Turnip mosaic virus
Source: Breed Sci. 2024 Aug 27;74(4):354–65. doi: 10.1270/jsbbs.24015 (PMC11769589; doi:10.1270/jsbbs.24015)
Supplement: Supplementary file 2 — Supplemental Tables [file 74_354_s2.pdf]

doi: 10.1270/jsbbs.24015

Supplemental Table 1 List of plant material accessions in this study

Accession	Variety name[a]	Source country
C101	Hatana 4 gou	Japan
C102	Thiba wase	Japan
C103	Wase zairai	Japan
C104	Enuma zairai	Japan
C105	Hatana 3gou	Japan
C107	Wasena (Yamagata)	Japan
C108	Wasenatane	Japan
C109	Miyagiwase	Japan
C110	Akatukatsukawase	Japan
C112	Enuma zairai	Japan
C120	(Kew-2)	UK
C121	n/a	USA
C123	n/a	USA
C137	Miyagiwase	Japan
C142	B.S.H-30	India
C144	B.S.H-40	India
C145	Yellow	India
C146	n/a	USA
C147	n/a	Canada
C149	n/a	Canada
C151	Hua-zi-you-cai	China
C152	Chugoku -saishin	Japan
C155	n/a	Canada
C220	Matsushima shin2gou	Japan
C240	Hakushokuhoutouren(B.pekinensis Rupr.)	Japan
C241	Kashinhakusai(B.pekinensis Rupr.)	Japan
C242	Aithihakusai(B.pekinensis Rupr.)	Japan
C243	Kenshinhakusai(B.pekinensis Rupr.)	Japan
C252	Hiratsuka 1 gou(B.pekinensis Rupr.)	Japan
C253	Kekkyu chiifuhakusai(B.pekinensis Rupr.)	Japan
C256	subsp. pekinensis	Thailand
C333	subsp. chinensis, Yukina	Japan
C334	Mana(B.chinensis L.)	Japan
C335	Shigatsu shirona (B.chinensis L.)	Japan
C336	Shakushina (B.chinensis L.)	Japan
C337	Maruba komatsuna (B.chinensis L.)	Japan
C338	Maruba mibuna (B.chinensis L.)	Japan
C339	Chitose kyou mizuna (B.chinensis shiro	Japan

C430	Tokinasi shirokokabu (B.rapa)	Japan
C455	Shyogoinkabu (B.rapa)	Japan
C464	n/a	New Zealand
C465	B.rapa	Egypt
C466	n/a	Egypt
C468	n/a	New Zealand
C470	Oguni-kabu	Japan
C471	B.rapa Sylvestris(L) Janchen	Spain
C472	Atsumi-kabu	Japan
C473	Hijiori-kabu	Japan
C474	Terauchi-kabu	Japan
C475	Miyazawa-kabu	Japan
C476	Gobouno-kabu	Japan
C477	n/a	n/a
C478	Toyama-kabu	Japan
C479	n/a	Korea
C482	Akane-kabu, N.783121	Japan
C483	Tsurayuki-kabu, N.793121	Japan
C503	var. toria	India
C505	var. toria	India
C506	var. toria	India
C507	var. toria, Itsa	India
C508	var. toria, Sangam	India
C509	var. toria, Assam Selection	India
C560	n/a	n/a
C632	var. sarson, T 74	Sweden
C633	var. sarson, T 87	Sweden
C634	var. sarson, USAD No. 180411	India
C635	var. sarson, USAD No. 217931	Pakistan
C636	var. sarson	Japan
C637	var. sarson	Japan
C642	n/a	India
C646	var. sarson	India
C647	n/a	India
C648	var. sarson	India
C651	var. sarson	India
C652	var. sarson	India
C653	var. sarson	India
C654	var. sarson	India
C655	var. sarson	India

C656	var. sarson	India
C663	var. sarson, CuIt-21	India
C664	var. sarson, COMPT-IV	India
C665	var. sarson, P.H.-28	India
C666	n/a	n/a
C667	var. sarson, DYS-1	India
C668	var. sarson, DYS-2	India
C669	var. sarson, DBS-2	India
C670	var. sarson, DBS-1	India
C701	n/a	Turkey
C702	n/a	Turkey
C703	n/a	Turkey
C705	n/a	n/a
C801	n/a	n/a
CR-seiga	Chinese Cabbage	Japan
Gokurakuten	Perviridis	Japan
Harusakari	Chinese Cabbage	Japan

[a]n/a=not applicable

Supplemental Table 2 List of Turnip mosaic virus isolates used in this study

MAFF no.[a]	Isolate name[b]	Isolated source plant species
260135	F2	Komatsuna
715062	F1	Rapeseed
104047	15	Chinese cabbage
715054	Ra-2	Radish
260136	My4	Turnip
260133	Tu-T10	Radish
260134	Tu-Ra	Radish
260137	Tu-190-1	Iceland poppy
260228	Tu-Rico	Leucocoryne
715027	n/a	Radish
715066	n/a	Stock (Matthiola incana)

[a] For details, refer to https://www.gene.affrc.go.jp/databases-micro_search.php

[b] n/a=not applicable

Supplemental Table 3 Primer list for cDNA synthesis of eIF4E and eIFiso4E

Gene	Forward primer (5'- 3')	Reverse primer (5'- 3')	PCR condition
eIF4E.a	ATGGCGGTAGAAGACACACTCAAGCC	TCAGGCAGTGTAAGCGCTCTTTGC	94°C, 2m; 98°C, 10s, 66°C, 30s, 68°C, 21s (35); 4°C
eIF4E.c	ATGGCGGTAGAAGACACTTCCAAGCC	TCAAGCGGTGTAAGCGCTCTTCG	94°C, 2m; 98°C, 10s, 66°C, 30s, 68°C, 21s (35); 4°C
eIFiso4E.a	ATGGCGACAGAGGATGT	TCAGACAGTGAACCGAGT	94°C, 2m; 98°C, 10s, 55°C, 30s, 68°C, 15s (35); 4°C
eIFiso4E.c	ATGGCGACAGAGGATGT	TCAGACACTAAATCGAC	94°C, 2m; 98°C, 10s, 55°C, 30s, 68°C, 15s (35); 4°C

Supplemental Table 4 Primer list for synthesis of TuMV P1 and VPg gene region

P1	MAFF no.[a]	Forward primer (5'- 3')	Reverse primer (5'- 3')	PCR condition
	104047	TCGTTATCAAAGCAATCACCAACAG	CGCCGCACTCAGTAACATCT	94°C, 2m; 98°C, 10s, 55°C, 30s, 68°C, 30s (35); 4°C
	260137	AGCAAACACAAATCTCTCGAAGC	GTTCACGGTTGTCACCTCGA	94°C, 2m; 98°C, 10s, 58°C, 30s, 68°C, 30s (35); 4°C
	715066	TCGTTATCAAAGCAATCACCAACAG	CGCCGCACTCAGTAACATCT	94°C, 2m; 98°C, 10s, 60°C, 30s, 68°C, 30s (35); 4°C
	715027	TCGTTATCAAAGCAATCACCAACAG	CGCCGCACTCAGTAACATCT	94°C, 2m; 98°C, 10s, 60°C, 30s, 68°C, 30s (35); 4°C
	715062	AGCAAACACAAATCTCTCGAAGC	GTTCACGGTTGTCACCTCGA	94°C, 2m; 98°C, 10s, 60°C, 30s, 68°C, 30s (35); 4°C
	260135	AGCAAACACAAATCTCTCGAAGC	GTTCACGGTTGTCACCTCGA	94°C, 2m; 98°C, 10s, 60°C, 30s, 68°C, 30s (35); 4°C
	260136	TCGTTATCAAAGCAATCACCAACAG	CGCCGCACTCAGTAACATCT	94°C, 2m; 98°C, 10s, 58°C, 30s, 68°C, 30s (35); 4°C
	715054	TCGTTATCAAAGCAATCACCAACAG	CGCCGCACTCAGTAACATCT	94°C, 2m; 98°C, 10s, 60°C, 30s, 68°C, 30s (35); 4°C
	260134	AGCAAACACAAATCTCTCGAAGC	GTTCACGGTTGTCACCTCGA	94°C, 2m; 98°C, 10s, 60°C, 30s, 68°C, 30s (35); 4°C
	260228	GGATGGCADCAGTYRCATT	ACATCRAGTCCTGTRTAACACGT	94°C, 2m; 98°C, 10s, 58°C, 30s, 68°C, 30s (35); 4°C
	260133	TCGTTATCAAAGCAATCACCAACAG	CGCCGCACTCAGTAACATCT	94°C, 2m; 98°C, 10s, 58°C, 30s, 68°C, 30s (35); 4°C
**VPg**	**MAFF no.[a]**	**Forward primer (5'- 3')**	**Reverse primer (5'- 3')**	**PCR condition**
	104047			
	260137			
	715066			
	715027			
	715062			
	260135	GAGGCAGTTCACCATCAAAACAC	GTGTTTTTAATCACGAACTCACCATGTC	94°C, 2m; 98°C, 10s, 58°C, 30s, 68°C, 30s (35); 4°C
	260136			
	715054			
	260134			
	260228			
	260133			

[a] For details, refer to https://www.gene.affrc.go.jp/databases-micro_search.php

Supplemental Table 5 NCBI accesion number list of identified sequence of P1 and VPg of TuMV

Sequnce name	NCBI accession number
MAFF104047_P1_ORF_only	OP730344
MAFF260137_P1_ORF_only	OP730345
MAFF715066_P1_ORF_only	OP730346
MAFF715027_P1_ORF_only	OP730347
MAFF715062_P1_ORF_only	OP730348
MAFF260135_P1_ORF_only	OP730349
MAFF260136_P1_ORF_only	OP730350
MAFF715054_P1_ORF_only	OP730351
MAFF260134_P1_ORF_only	OP730352
MAFF260228_P1_ORF_only	OP730353
MAFF260133_P1_ORF_only	OP730354
MAFF715054_VPg_ORF_only	OP730355
MAFF715062_VPg_ORF_only	OP730356
MAFF715066_VPg_ORF_only	OP730357
MAFF260136_VPg_ORF_only	OP730358
MAFF260133_VPg_ORF_only	OP730359
MAFF104047_VPg_ORF_only	OP730360
MAFF715027_VPg_ORF_only	OP730361
MAFF260228_VPg_ORF_only	OP730362
MAFF260137_VPg_ORF_only	OP730363
MAFF260135_VPg_ORF_only	OP730364
MAFF260134_VPg_ORF_only	OP730365

Supplemental Table 6 dCAPS markers for amino acid substitutions in eIF4E and eIF(iso)4E

SNP	Forward primer (5'- 3')	Reverse primer (5'- 3')	Enzyme	Buffer (10X)
eIF4E.a-12	AGAAGACACACTCAAGCCTAATGTA	ACTCCAGAACTCCTCGACGGTGG	AluI	L
eIF4E.a-21	AGAAGACACACTCAAGCCTAATGTA	ACTCCAGAACTCCTCGACGGTGG	PstI	H
eIF4E.a-40	AAGGCGACGATGCCGAGGAAGGAGCGA	ACTCCAGAACTCCTCGACGGTGG	HaeIII	M
eIF4E.a-112	CCACTTAGGTTCAATATTGTGTTTGAAT	CCACCGTCGAGGAGTTCTGGAG	HinfI	H
eIFiso4E.a-108	CTGAGTGTGCTAATGGGGGAAAGTGGACAT	CTTCTTCCCAATACCCATCAGAACAGC	NdeI	H
eIF4E.c-35	ATGGCGGTAGAAGACACTTCCAAGCC	TTCGCCGCCGGCGATCTCTCCTTCGTCA	HincII	M
eIF4E.c-45	GAGGAAGGAGAGATCGCCGGCGGCTAA	GAACAGACTCCAGAACTCCTCGACGGTGA	DdeI	K
eIF4E.c-105	GGAGTTCTGGAGTCTGTTCAATAACCTGA	CAAACTGTTCTCCAATCAACGCAAGC	DdeI	K
eIF4E.c-201	CCTTTGATGTTGCAGGTGAGCATTGGAA	TCAAGCGGTGTAAGCGCTCTTCG	MboII	L
eIFiso4E.c-36	TCCTCACAAGCTCGAAAGAAAGTGGAGT	TTTGAACATGTGAATGTCAGCGTTGGGG	AluI	L
eIFiso4E.c-52	GAAACCCAAGCAAGGCGCCGCCTGGGGAT	TTGAACATGTGAATGTCAGCGTTGGAT	MboI	K
eIFiso4E.c-80	GAAACCCAAGCAAGGCGCCGCCTGGGGAT	TTGAACATGTGAATGTCAGCGTTGGAT	EcoRV	H
eIFiso4E.c-150	TCTGTGGTGTGGTTGCTAGTGTCCGGC	TCAGACACTAAATCGAC	HaeIII	M

Supplemental Table 7 Recombination events in turnip mosaic virus P1 genome detected by recombination detecting programs

TuMV (MAFF no.)[a]	Minor/Major parent	Type of recombination[b]	Software[c]	*P*-value[d]
260137	LC537477.1/AB362513.1	World-B × Asian-BR	RGBMC**S**3	3.29E-15
715062	KC119186.1/AB362513.1	World-B × Asian-BR	**R**GMS3	3.35E-08
260135	LC537541.1/AB362513.1	World-B × Asian-BR	RGB**M**CS3	1.95E-11
260134	LC537541.1/AP017884.1	World-B × Asian-BR	RGBMC**S**	2.49E-15

[a] Recombination events detected in the turnip mosaic virus genomes by RDP from the aligned sequences of their genomes.

[b] Phyogenetic groups of minor/major parental isolates.

[c] Recombination isolates identified by the recombination detecting programs, containing M (MAXCHI), C (CHIMAERA), S (SISCAN), G (GENECONV), B (BOOTSCAN) programs in RDP. The program which gave smallest *P*-value among the other detected programs is shown in bold font.

[d] The smallest *P*-values of the programs shown in bold font were reported in a range among all isolates which share the same recombination event.

Supplemental Table 8 Clusters of eleven TuMV isolates according to phylogenic and recombination analysis of CP and P1 sequence of TuMV

MAFF no.[a]	CP sequence[b]	VPg sequence[c]	P1 sequence[d]	Cluster[f]
715062	Asian-BR	Asian-BR	World-B × Asian-BR[e]	1
260135	Asian-BR	Asian-BR	World-B × Asian-BR[e]	1
260137	Asian-BR	World-B	World-B × Asian-BR[e]	2
260134	Asian-BR	World-B	World-B × Asian-BR[e]	2
260228	Basal-B	World-B	Basal-B	3
715027	World-B	Asian-BR	World-B	4
260136	World-B	World-B	World-B	5
715054	World-B	World-B	World-B	5
104047	World-B	World-B	World-B	5
715066	World-B	World-B	World-B	5
260133	World-B	World-B	World-B	5

[a] For details, refer to https://www.gene.affrc.go.jp/databases-micro_search.php

[b] The evleven TuMV isolates were clustered into Asian-BR, Basal-B, and World-B group according to the phylogenic analysis of CP sequence of TuMV.

[c] The evleven TuMV isolates were clustered into Asian-BR and World-B group according to the phylogenic analysis of VPg sequence of TuMV.

[d] The seven TuMV isolates without recombinants detected were clustered into Basal-B and World-B group according to the phylogenic analysis of P1 sequence of TuMV.

[e] Recombinantion event detected in TuMV(715062), TuMV(260135), TuMV(260137) and TuMV(260134)

[f] Three clusters of eleven TuMV isolates according to phylogenic and recombination analysis of CP and P1 sequence of TuMV.

Supplement Table 9 Nucleotide/amino acid polymorphisms in eIF4Es and eIF(iso)4Es among fifteen accessions of *B. rapa*

nucleotide acid variants	*eIF4E.a*												
	30	34	62	116	119	300	335	444	525	540	564	588	663
C107	T	G	C	C	C	C	A	C	A	G	T	A	A
C108	C	C	T	C	T	T	A	C	A	G	T	G	C
C109	T	G	T	C	T	T	A	C	A	G	T	G	C
C110	T	G	C	C	C	C	G	C	G	G	G	G	C
C112	C	C	T	C	T	C	A	C	A	G	T	A	C
C121	C	C	T	C	T	C	G	C	A	G	T	A	C
C123	C	C	T	C	T	T	A	C	A	G	T	A	C
C137	C	C	T	C	T	T	A	C	A	G	T	A	C
C220	T	G	C	C	C	C	G	T	G	G	G	G	C
C253	C	C	C	C	T	T	A	C	A	G	T	A	A
C470	C	C	T	T	T	C	A	C	A	T	A	G	C
C634	T	G	C	C	C	C	G	C	G	G	G	G	C
C636	T	G	C	C	C	C	G	C	G	G	G	G	C
Gokurakuten	T	G	T	C	T	T	A	C	A	G	T	A	C
Harusakari	C	C	T	C	T	T	A	C	A	G	T	G	C

nucleotide acid variants	*eIF(iso)4E.a*						
	79	235	309	323	330	420	426
C107	G	A	C	T	T	C	G
C108	G	A	C	T	T	C	G
C109	G	A	C	T	T	C	G
C110	G	A	C	T	T	C	G
C112	G	A	C	T	T	C	G
C121	G	A	C	T	T	C	G
C123	G	T	C	T	T	C	G
C137	G	A	C	T	T	C	G
C220	G	A	C	T	T	C	G
C253	G	A	C	T	T	C	G
C470	G	A	C	T	T	C	G
C634	C	A	G	A	C	T	C
C636	C	A	G	A	C	T	C
Gokurakuten	G	A	C	T	T	C	G
Harusakari	G	A	C	T	T	C	G

nucleotide acid variants	*eIF4E.c*															
	104	105	133	134	162	198	273	274	314	360	393	399	429	462	495	602
C107	T	T	A	C	C	C	C	T	A	G	C	T	G	T	C	A
C108	C	T	G	G	C	T	T	T	A	A	T	T	G	C	T	G
C109	T	T	A	C	C	C	C	T	A	G	C	T	G	T	C	A
C110	T	T	A	C	C	C	C	T	A	G	C	T	G	T	C	A
C112	T	T	A	C	C	C	C	T	A	G	C	T	G	T	C	A
C121	C	C	A	C	C	C	C	G	A	A	T	A	A	C	T	G
C123	C	T	G	G	G	T	T	T	A	G	T	T	G	C	C	A
C137	T	T	A	C	C	C	C	T	A	G	C	T	G	T	C	A
C220	T	T	A	C	C	C	C	T	A	G	C	T	G	T	C	A
C253	C	T	G	G	C	T	T	T	A	A	T	T	G	C	T	G
C470	C	T	G	G	C	T	T	T	A	G	C	T	G	T	C	A
C634	C	T	G	G	C	T	C	T	G	G	C	T	G	C	T	A
C636	C	T	G	G	C	T	C	T	G	G	C	T	G	C	T	A
Gokurakuten	C	T	G	G	C	T	T	T	A	A	T	A	A	C	T	A
Harusakari	T	T	A	C	C	C	C	T	A	G	C	T	G	T	C	A

nucleotide acid variants	*eIF(iso)4E.c*					
	106	155	239	381	449	546
C107	T	C	C	A	C	C
C108	T	C	T	G	C	C
C109	T	C	T	G	A	C
C110	T	C	T	G	C	C
C112	T	C	T	G	C	C
C121	T	C	C	G	C	C
C123	T	C	C	G	A	T
C137	T	C	C	G	C	C
C220	T	C	T	G	C	C
C253	T	C	T	G	C	C
C470	T	C	C	A	C	C
C634	C	T	C	G	A	T
C636	C	T	C	G	A	T
Gokurakuten	T	C	C	A	C	C
Harusakari	T	C	T	G	C	C

amino acid variants	*eIF4E.a* 12	21	40	112	*eIF(iso)4E.a* 27	79	108	*eIF4E.c* 35	45	92	105	201	*eIF(iso)4E.c* 36	52	80	150
C107	A	A	T	Y	D	T	F	V	T	S	K	K	F	A	T	P
C108	P	V	I	Y	D	T	F	A	G	S	K	R	F	A	I	P
C109	P	V	I	Y	D	T	F	V	T	S	K	K	F	A	I	Q
C110	A	A	T	C	D	T	F	V	T	S	K	K	F	A	I	P
C112	P	V	I	Y	D	T	F	V	T	S	K	K	F	A	I	P
C121	P	V	I	C	D	T	F	A	G	A	K	R	F	A	T	P
C123	P	V	I	Y	D	S	F	A	G	S	K	K	F	A	T	Q
C137	P	V	I	Y	D	T	F	V	T	S	K	K	F	A	T	P
C220	A	A	T	C	D	T	F	V	T	S	K	K	F	A	I	P
C253	P	A	I	Y	D	T	F	A	G	S	K	R	F	A	I	P
C470	P	V	I	Y	D	T	F	A	G	S	K	K	F	A	T	P
C634	A	A	T	C	H	T	Y	A	G	S	R	K	L	V	T	Q
C636	A	A	T	C	H	T	Y	A	G	S	R	K	L	V	T	Q
Gokurakuten	A	V	I	Y	D	T	F	A	G	S	K	K	F	A	T	P
Harusakari	P	V	I	Y	D	T	F	V	T	S	K	K	F	A	I	P

Supplement Table 10 Amino acid substitutions in eIF4E/eIFiso4E based on dCAPs analysis

Plant	eIF4E.a				eIFiso4E.a	eIF4E.c				eIFiso4E.c			
accession	12	21	40	112	108	35	45	105	201	36	52	80	150
**C121**	P	V	I	C	F	A	G/T	K	R	F	A	T	P
**C123**	P	V	I	Y	F	A	G	K	K	F	A	T	P/Q
**C146**	A	A	I	Y	F/Y	A	G	K	K/R	F	A	T	P/Q
**C149**	P	V	I	Y	F/Y	V/A	G/T	K	K/R	F	A	T	P/Q
**C155**	P	V	I	Y	F	A	T	K	K	F	A	T	Q
**C252**	A	A	T	C	F	V/A	G/T	K	K/R	F	A	I	P
**C464**	P	V	I	Y	F/Y	A	G	K	K	F	A	T	P
**C465**	P	V	I	Y	F/Y	A	G	K	K	F	A	T	Q
**C466**	P	V	I	Y	F	A	G	R	K	F	A	T	P/Q
**C471**	P	V	I	Y	F	A	G	K	K	F	A	T	P
**C473**	P	V	T	C	F	A	T	K	K	F	A	T	Q
**C482**	P	V	I	Y	Y	V	T	K	K	F	A	T	P/Q
**C483**	P	V	I	Y	Y	V/A	G/T	K	K	F	A	I/T	P/Q
C101	P	V	I	Y	F	V	T	K	K	F	A	I/T	P
C102	P	V	I	Y	F	V/A	G/T	K	K	F	A	I/T	P
C103	P	V	I	Y	F	A	G	K	R	F	A	I	P
C104	P	V	I	Y	F/Y	V	T	K	K	F	A	T	P/Q
C105	P	V	I	Y	F/Y	V	T	K	K	F	A	T	Q
C107	A	A	T	Y	F	V	T	K	K	F	A	T	P
C108	P	V	I	Y	F	V/A	G/T	K	K/R	F	A	I/T	P
C109	P	V	I	Y	F	V	T	K	K	F	A	I	Q
C110	A	A	T	C	F	V	T	K	K	F	A	I	P
C112	P	V	I	Y	F	V	T	K	K	F	A	I	P
C120	A	A	I	C	F	A	G	K	K	F	A	I	P
C137	A/P	A/V	I	Y	F	V	T	K	K	F	A	I/T	P/Q
C142	A/P	A/V	I	Y	Y	A	G/T	K	K	F	A/V	T	Q
C144	A/P	A	I	Y	F/Y	V/A	G/T	K	K	L	V	T	Q
C145	A	A	T	C	Y	A	G	R	K	L	V	T	Q
C147	A/P	A/V	I	Y	F/Y	A	T	K	K	F	A	T	Q
C151	P	V	I	Y	F/Y	V/A	G/T	K	K	F	A	T	P
C152	P	V	I	Y	Y	V	T	K	K	F	A/V	I/T	P/Q
C220	A	A	T	C	F	V	T	K	K	F	A	I	P
C240	P	V	I	Y	F/Y	V/A	G/T	K	K	F	A	I	P
C241	P	V	I	Y	F	V	T	K	K	F	A	I/T	P
C242	A	A	T	C	F	V	T	K	K	F	A	I/T	P
C243	P	V	I	Y	F	V	T	K	K	F	A	I	P
C253	A/P	A/V	I	Y	F	V/A	G/T	K	K/R	F	A	I	P
C256	A/P	A/V	I	Y	F/Y	V/A	G/T	K	K/R	F	A	I/T	P
C333	A	A/V	I	Y	F	V/A	G/T	R	K	F	A	T	P
C334	P	V	I	Y	F	V	T	K	K	F	A	T	P
C335	P	V	I	Y	F	V	T	K	K	F	A	I/T	P/Q
C336	P	V	I	Y	F/Y	V/A	G/T	K	K	F	A	T	P
C337	A/P	A/V	I	Y	F	V	G/T	K	K	F	A	I/T	P
C338	P	V	I	Y	F	V	T	K	K	F	A	T	P
C339	P	V	I	Y	F/Y	V	T	K	K	F	A	I/T	P
C430	P	V	I	Y	F/Y	V	T	K	K	F	A	I/T	P
C455	P	V	I	Y	F	V	T	K	K	F	A	T	P
C468	P	V	I	Y	F	A	G	K	K/R	F	A	I/T	P
C470	P	V	I	Y	F	V/A	G/T	K	K	F	A	T	P
C472	A	A	I	C	F	A	G/T	K	K/R	F	A	T	P/Q
C474	P	V	I	Y	F	V	T	K	K	F	A	I/T	P
C475	P	V	I	Y	Y	V	T	K	K	F	A	I/T	P/Q
C476	P	V	I	Y	F	V/A	G/T	K	K	F	A	T	P/Q
C477	P	V	I	Y	F	V	T	K	K	F	A	T	Q
C478	P	V	I	Y	F	V	T	K	K	F	A	I/T	P
C479	A	V	T	C	F	A	T	K	K/R	F	A	T	Q
C503	P	V	I	Y	Y	V/A	G/T	K	K	L	A	T	Q
C505	A	A	I	Y	F	V/A	G/T	K	K	F	A	T	Q
C506	A	A	I	Y	Y	V	T	K	K	F	A	I/T	P/Q
C507	A	A	I	Y	Y	V/A	G/T	K	K	F	A	T	Q
C508	A	A	I	Y	Y	V	T	K	K	F	A	T	P/Q
C509	A	A	I	Y	Y	V/A	G/T	K	K	L	V	T	Q
C560	A	A	I	Y	F	V/A	G/T	K	K	L	A	T	Q
C632	A	A	T	C	Y	A	G	R	K	L	V	T	Q
C633	A	A	T	C	F/Y	A	G	R	K	L	V	T	Q
C634	A	A	T	C	Y	A	G	R	K	L	V	T	Q
C635	A	A	T	C	Y	A	G	R	K	L	V	T	Q
C636	A	A	T	C	Y	A	G	R	K	L	V	T	Q
C637	A	A	T	C	Y	A	G	R	K	L	V	T	Q
C642	A	A	T	C	Y	A	G	R	K	L	V	T	Q
C646	A	A/V	I	Y	Y	V	T	K	K	F	A	I/T	P/Q
C647	A	A	I	Y	F	V/A	G/T	K	K	F	A	T	Q
C648	A	A	I	Y	Y	V/A	G/T	K	K	L	A	T	P/Q
C651	A	A	T	C	Y	A	G	R	K	L	V	T	Q
C652	A	A/V	I	Y	Y	V/A	G/T	K/R	K	F	A	T	P
C653	A	A	T	C	Y	A	G	R	K	L	V	T	Q
C654	P	A	T	C	F	A	G	R	K	L	V	T	Q
C655	A	A	T	C	Y	A	G	R	K	L	V	T	Q
C656	P	A	I	Y	F	V/A	G/T	K	K	L	V	T	Q
C663	P	A	I	Y	F	V	T	K	K	L	V	T	Q
C664	A	A	I	Y	Y	V/A	G/T	R	K	L	A	I/T	Q
C665	A	A	I	Y	F	V	T	K	K	L	A	I/T	Q
C666	A	A	I	Y	F	V	T	K	K	F	A	T	P
C667	A	A	T	C	F/Y	A	G	R	K	L	V	T	Q
C668	A	A	T	C	F/Y	A	G	R	K	L	V	T	Q
C669	A	A	T	C	F/Y	A	G	R	K	L	V	T	Q
C670	A	A	T	C	F/Y	A	G	R	K	L	V	T	Q
C701	P	A	T	C	F/Y	V	T	K	K	F	V	T	P
C702	P	V	I	C	F	V	G	K	K	F	A	T	P
C703	P	A	T	C	F/Y	V	T	K	K	F	A/V	T	P
C705	P	A/V	I	C	Y	V	T	K	K	F	A	T	P
C801	P	V	I	Y	Y	A	G	K	K	F	A	T	Q
CR-seiga	P	V	I	Y	F	V	T	K	K	F	A	I	P
Gokurakuten	A	V	I	Y	F	A	G	K	K	F	A	T	P
Harusakari	P	V	I	Y	F	V	T	K	K	F	A	I	P
